# Annotations

**Published:** 1905-04-01

**Authors:** 


### ANNOTATIONS.

### Hospitals and Medical Schools.

Sir Edward Fry's committee in its report submitted that the distinction between the hospital and the school should in every case be drawn not only definitely and exactly, but with such clearness that it may be understood by the general public, so that no question may arise as to the destination and application of moneys contributed, whether by the King's Fund or from any other source. This recommendation has speedily borne fruit. Lord Stanley, the treasurer of the London Hospital, has written to the *Times* to say that the London Hospital has opened a discretionary fund to be used in whole or in part at the discretion of the committee for either the hospital or the school. He adds that within a week of this fund being started the amount transferred to it by London Hospital subscribers was more than twice the amount they contributed to the school. This wise solution of a question which has caused some unrest in the minds of the public and hospital subscribers generally reflects the greatest credit on the committee of the London Hospital and all associated with its management. Their action is the more praiseworthy from the circumstance that in every copy of the accounts of the London Hospital the payments heretofore made to the Medical College have appeared as a separate item, and such payments have, moreover, on more than one occasion had the unanimous approval of the Governors of the hospital. We have more than once pointed out that the course adopted by the London Hospital would at once solve the difficulty which has arisen, and we hope that the same course may be followed by every metropolitan medical school without delay. Then every ground for criticism in regard to this matter will be removed; public confidence will be restored, and the hospitals with medical schools cannot fail to reap substantial benefits in consequence. Mr. Sydney Holland has fought this question hard, and he is to be congratulated upon his prompt action in the matter.

### The Medical Graduates' College and Polyclinic.

The annual general meeting of the Medical Graduates' College and Polyclinic, which was held on March 24th, affords a convenient opportunity to review the progress that has been made since the Polyclinic was first established some six years ago. According to the Report for 1904, the members and subscribers now number nearly nine hundred, and though this is below the ambition of the founders of the college it is yet a substantial total. As the educational and clinical opportunities afforded by the Polyclinic become more widely known the appreciation of them will doubtless grow. It is all to the good that practitioners can find on any afternoon in the week a demonstration of clinical cases selected as examples of the most approved methods of diagnosis and treatment. During last year it appears that more than twelve hundred cases were shown in this manner; and when it is remembered that the great majority of these were chosen because they illustrated important points either of theory or practice, the value of the clinical opportunities offered by the Polyclinic must be ranked at a high level. The council maintains its policy in reference to fees, and for the small charge of one guinea all the advantages of the Polyclinic are open to any registered practitioner for the term of one year. It is satisfactory to note an improvement in the state of the finances. In former years the balance sheet invariably showed a serious deficit, but after the anxious position thus created the new council has proceeded on sounder business principles; and hence, for the first time in the history of the Polyclinic the year's expenditure has been kept within the year's income. Upon this all the friends of the college may be congratulated. As an important part of the scheme of post-graduation teaching in the metropolis the Polyclinic deserves encouragement and support, and the fact that it cultivates a platform not restricted to one special school or sect ought to commend it to all who desire to improve the position of London as a centre of medical education and influence.

### Medical Doctrine in the Lay Press.

The mistakes of the ordinary journalist when dealing with medical affairs form a familiar and often also an amusing experience. To an uninformed public there is probably but little damage done by these mistakes, as each individual, however replete with "views" regarding disease and its treatment so long as he enjoys good health, generally seeks technical direction at the first suggestion of real illness. The position is somewhat different when a medical practitioner enters the columns of the lay press and proceeds to discuss questions of treatment and diagnosis. The fact that the writer of such an article appears in a non-medical publication is largely taken by the public to mean that he is a recognised exponent of professional views, and hence his opinions are apt to receive in lay circles a degree of respect and attention far in excess of their merits. In a recent number of a leading review a medical practitioner has written an article on appendicitis and the causes of this disease, in which he announces conclusions which he himself would admit are not generally adopted by the medical profession; and it is impossible to avoid asking what good end is served by such a policy. Appropriate enough as a subject for a discussion in the medical press or in a professional society, it is singularly out of place in columns that appeal to the general public. The audience obviously has not the technical information or experience necessary to judge or decide the issue, and except as an advertisement for the writer nothing but harm can follow such an article. There are occasions on which it is the duty of the profession to appeal through the lay press to the general public, so that the health of the community may be safeguarded, and when this is done by an appropriate authority the service rendered to the body politic is a considerable one. But to attempt to discuss doubtful points of medical doctrine in the lay journals is conducive neither to the discovery of truth nor to the dignity of the profession.

